# Understanding coping strategies during pregnancy and the postpartum period: a qualitative study of women living with HIV in rural Uganda

**DOI:** 10.1186/s12884-017-1321-9

**Published:** 2017-05-08

**Authors:** Scholastic Ashaba, Angela Kaida, Bridget Frances Burns, Kasey O’Neil, Emma Dunkley, Christina Psaros, Jasmine Kastner, Alexander C. Tsai, David R. Bangsberg, Lynn T. Matthews

**Affiliations:** 1 Department of Psychiatry, Mbarara University of Science and Technology, Mbarara, Uganda; 2 Faculty of Health Sciences, Simon Fraser University, Burnaby, Canada; 3 MGH Global Health, Massachusetts General Hospital, Boston, MA USA; 4 Department of Psychiatry, Massachusetts General Hospital, Boston, MA USA; 5 Harvard Medical School, Boston, MA USA; 6 Research Institute McGill University Health Centre Montreal, Montreal, Canada; 7 Division of Infectious Diseases, Department of Medicine, Massachusetts General Hospital, Boston, MA USA

**Keywords:** Women, HIV, Pregnancy, Postpartum, Coping strategies, Rural Uganda

## Abstract

**Background:**

In sub-Saharan Africa, 58% of adults living with HIV are women. In Uganda, HIV prevalence is 8.3% for women compared to 6.1% for men. Access to antiretroviral therapy (ART) and prevention of mother to child transmission (PMTCT) programs have enabled women living with HIV (WLWH) to have children with minimal risk of perinatal transmission. Nevertheless, pregnant WLWH face many challenges. We explored women’s perceptions of how they cope with the challenges of pregnancy and the postpartum period as HIV-infected women.

**Methods:**

We conducted semi-structured interviews with postpartum WLWH accessing ART who had a pregnancy within 2 years prior to recruitment between February–August, 2014. Childbearing associated stressors and coping strategies were discussed. We used content analysis to identify major themes and NVivo 10 software facilitated data analysis.

**Results:**

Twenty women were interviewed with median age 33 (IQR: 28–35) years, CD4 cell count 677 cells/mm^3^ (IQR: 440–767), number of live births 4 (IQR: 2–6), and number of living children 3 (IQR: 2–4.3). We summarize five identified coping strategies within a socio-ecological framework according to Bronfenbrenner’s Ecological Model. Coping strategies on the individual level included acceptance of self and HIV status, and self-reliance. On the interpersonal level, participants reported coping through support from partners, family, and friends. On the organizational level, participants reported coping through HIV-related healthcare delivery and system supports. At the community level, women reported coping through support from church and spirituality.

**Conclusions:**

The results highlight coping strategies used by WLWH to manage the myriad challenges faced during pregnancy and the postpartum period. Intervention programs for WLWH must emphasize psychosocial care and incorporate strategies that address psychosocial challenges in the HIV care package in order to optimize well-being. Additionally policies that support networks of WLWH should be put in place and funding support should be provided through existing funding mechanisms in order to respond to the needs and challenges of WLWH. Programmes that support WLWH for economic empowerment and improved livelihoods should be strengthened across all regions in the country.

**Electronic supplementary material:**

The online version of this article (doi:10.1186/s12884-017-1321-9) contains supplementary material, which is available to authorized users.

## Background

Worldwide, HIV disproportionately affects women and in sub-Saharan Africa 58% of adults living with HIV are women [1]. The adult prevalence of HIV in Uganda is 7.3% overall; 8.3% for women compared to 6.1% among men [2]. Access to antiretroviral therapy (ART) and prevention of mother to child transmission (PMTCT) programs enable women living with HIV (WLWH) to have children with minimal risks to their health and perinatal transmission [3]. However, being HIV positive and pregnant remains a challenging experience for women [4, 5]

Pregnant WLWH experience psychosocial challenges during pregnancy and childbirth including emotional distress and fear of perinatal transmission [6, 7], stigma [8] lack of social support [9–11] , food insecurity [12] and poverty [7, 13], challenges related to disclosure of HIV status [14–19] , violence [20–22], and concerns about their own physical health and survival. These stressors increase women’s vulnerability and psychological distress which can further compromise their pregnancy and postpartum experience [23–26] and lead to a range of emotional and psychological problems including postpartum depression [22, 27–29].

Despite the myriad of challenges experienced by WLWH there is limited data exploring how women in resource poor settings cope with the challenges. Yet a strong relationship between coping and psychological wellbeing among people living with chronic illnesses including HIV has been reported [30]. According to Lazarus and Folkman [31], coping is a process that mediates the relationship between experiences and outcomes of psychological stress. In the process of coping, individuals use both cognitive and behavioral strategies to manage stressful situations. Coping helps individuals to manage emotional distress and the problems that cause psychological distress. Coping strategies used by individuals to manage emotional distress are known as emotion-focused coping strategies while those used by individuals to manage situations causing distress are known as problem-focused coping strategies [31]. Previous research shows that people living with HIV who employ positive coping can realize positive psychosocial and physical health outcomes including fewer physical symptoms, enhanced quality of life, less psychological distress, and higher self-esteem [32–36].

Active coping involves behavioural and cognitive efforts to actively confront the stressful situation and challenges using strategies like problem-solving, changing the mind set and seeking relevant information [25]. In addition, social support and coping may influence outcomes of chronic illnesses through promoting medication adherence [33, 37]. Positive strategies for coping help individuals manage emotional stress while also helping them to manage problems that cause psychological distress [31] .

In a previous Uganda AIDS Rural Treatment Outcome (UARTO) cohort study of women living with HIV accessing ART, we observed a high pregnancy incidence of 9.4/100 person years of follow-up, with nearly one-third of women becoming pregnant within 3 years of ART initiation [38]. In this cohort, 39% of women were depressed, with higher depression scores among pregnant and postpartum women [39] . While the rollout of Option B+ and other strategies to reduce perinatal transmission are important in supporting women to meet their reproductive goals and have uninfected children [40], supporting women’s mental and emotional health is also crucial to growing healthy families, communities, and populations. Understanding how WLWH cope with the challenges associated with pregnancy and the postpartum period is key to the development of effective interventions that can minimize psychological distress and support healthy pregnancy and postpartum outcomes. Using qualitative data from a study exploring experiences of depression among pregnant WLWH engaged in care, this paper explores women’s descriptions of how they cope with the challenges of pregnancy and postpartum period.

## Methods

### Study setting

We conducted this study in Mbarara, a rural town in Southwestern Uganda located approximately 270 km from Kampala, the capital city. Mbarara town has an estimated population of 195,013 people [41]. Adult HIV prevalence in the region is estimated at 10%, with women carrying a higher burden of infection [42] with a reported prevalence of 12% among women attending antenatal clinic in Mbarara hospital [42, 43] . All study participants were accessing care at a public HIV clinic within the regional referral hospital. The clinic offers comprehensive HIV care, including ART, free of charge to patients.

### Study participants and recruitment

Study participants were WLWH recruited from the Uganda AIDS Rural Treatment Outcomes (UARTO) cohort [43]. From 2005–2015, UARTO followed over 700 HIV-infected men and women of at least 18 years of age, on ART, receiving care at the local HIV clinic, and living within 60 km of the clinic site. Eligible participants for this qualitative sub-study were females, enrolled in the parent UARTO study; and experienced a pregnancy in the 2 years prior to recruitment. The primary aim was to explore experiences of depression among WLWH during pregnancy and postpartum. We used stratified random sampling to select eligible participants with a range of depression experiences based on their responses to the Hopkins Symptoms Checklist (HSCL-16) within the parent cohort study.

During a previous UARTO study visit, women were screened for depression symptom severity using a modified version of the HSCL-15 for depression. Based on previous studies using HSCL in Uganda, a 16th item was included: “Feeling like I don’t care about my health” [44]. Each of the 16 symptoms is scored on a 4-item Likert scale ranging from not at all (1), a little (2), quite a bit (3), to extremely (4), and the total depression severity score was calculated as the mean of the 16 items, with higher scores indicating greater depression symptom severity. The HSCL has been used to assess depression in general population samples and among people living with HIV in sub-Saharan African settings, with good reliability and contrast validity [45]. We considered a dichotomous measure of “probable depression”, defined as an HSCL-16 score > 1.75, which has been previously used as a positive screen for depression in the UARTO cohort study and other sites [39, 44, 46, 47].

We selected women with 4 response patterns on the Hopkins Symptoms Checklist (HSCL) scores collected in quarterly visits prior to, during, and after the referent pregnancy: (1) HSCL scores falling below 1.75 postpartum; (2) HSCL rising above 1.75 postpartum; (3) stably high (≥1.75) HSCL scores; and (4) stably low (<1.75) HSCL scores. Trained research assistants (RAs) contacted potential participants by phone, explained the purpose of the study, the benefits, and the risks of participating. If interested, the Research assistant (RA) scheduled an interview at a location and time chosen by the participant and the informed consent process took place on the interview day, ensuring voluntary participation, confidentiality and safety.

### Data collection

We conducted semi-structured interviews with twenty participants from February through August 2014 using a structured interview guide. Point of saturation was reached for our primary research question of women’s experiences of depression during the postpartum period. We developed the interview guide based on the literature of postpartum depression with input from both local and international experts with experience in reproductive and mental health. We used guidelines articulated by Huberman and Miles [48]. We piloted the interview guide with study staff, and colleagues to assess its clarity and content, and revised accordingly. We translated and back-translated the guide to ensure consistency.

Interviews lasted approximately one hour and explored women’s experiences during pregnancy and postpartum periods. The interview guide explored symptoms of depression and the effect on the pregnancy and postpartum experience of HIV positive women, participants’ thoughts and feelings about becoming pregnant, how their HIV status influenced their thoughts and feelings towards pregnancy, and their partner’s thoughts towards the referent pregnancy. The final section of the interview explored feelings and experiences of the participants following childbirth (see Additional file 1). The interview guide was piloted extensively prior to use. After piloting, some of the questions in the interview guide were dropped and others rephrased to improve clarity. Ugandan RAs trained in qualitative research methods and fluent in English and the local language conducted the interviews and were blinded to participants’ quantitative depression scores and patterns.

### Ethical considerations

All participants provided voluntary written informed consent at study enrollment. The Institutional Review Committee, Mbarara University of Science and Technology; the Partners Human Research Committee, Massachusetts General Hospital; and the Research Ethics Board of Simon Fraser University approved the study. We received clearance for conducting our study from the Uganda National Council for Science and Technology and from the Research Secretariat in the Office of the President.

A Ugandan psychiatrist (SA) trained research assistants to recognize signs and symptoms of depression. If the RAs noticed signs or symptoms of acute and severe distress, they were instructed to refer the participant to the Mental Health Clinic. Two participants used our referral protocol over the course of the study. RAs were intentionally blinded to the depression scores of the participants.

### Data analysis

Demographic information for each participant was collected from the UARTO cohort database. RAs translated and transcribed audio recordings of the interviews into English. Transcripts were reviewed by separate RAs and a psychiatrist (SA) to assess translation quality and fidelity. All research team members reviewed and discussed transcripts and developed a codebook (see Additional file 2) through an iterative process. The codebook guided the coding process, which was completed by two members of the research team. After coding a subset of four interviews, the two coders compared coding for four interviews to ensure coding reliability and to verify understanding of the codebook (Kappa statistic = 0.82) [49] after which they coded the rest of the interviews independently. After coding all interviews, the research team further discussed the emergent themes in the context of coding. We used thematic analysis to identify major themes and NVivo 10 software facilitated data analysis.

## Results

Forty-two participants were eligible to participate in the study and 20 were recruited, including 4 with falling HSCL scores postpartum, 6 with rising HSCL scores postpartum, 3 with stably high HSCL scores, and 7 with stably low HSCL scores. We aimed to recruit a similar number of participants in each category, however only 5 of 42 eligible participants had stably high HSCL scores.

Participants had a median age of 33 years (range 22 to 40) and 95% had achieved plasma HIV-RNA suppression. Women had a median of 3 living children and a median CD4 cell count of 677 cells/mm^3^ (Table 1).

**Table 1:** Socio-demographic characteristics of women living with HIV with pregnancy within 2 years prior to interview (*n* = 20)

Characteristic	Median [IQR]
Age (years)	33 [28, 35]
Time on ART (years)	2.3 [1.8,5.1]
Number of live births	4 [2, 6]
Number of living children	3 [2, 4]
Months between pregnancy outcome and interview	15 [7, 21]
Most recent CD4 cell count (cells/mm^3^)	677 [440,767]

### Challenges and coping

Women in our study described several challenges to being a pregnant HIV-positive woman, including lack of financial means to secure adequate food, shelter, and clothing; lack of support for financial and emotional needs; difficulties adhering to ART and navigating PMTCT guidelines; and internalized HIV-related stigma. In the face of tremendous challenges, the coping strategies that women employed to manage hardships were discussed.

We summarize the five identified coping strategies within a more general socio-ecological framework [50]. On the individual level, coping strategies included (1) acceptance of self and HIV status, and (2) self-reliance. On the interpersonal level, participants reported coping through (3) financial, emotional, and social support from partners, family, and friends. On the organizational level, participants reported coping through (4) HIV-related healthcare delivery and system supports. On the community level, women reported gleaning (5) support from the church community and spirituality.

We discuss each of these five themes with a view to informing interventions to support pregnant and postpartum WLWH to overcome psychosocial challenges.

1. Individual level: Acceptance of HIV serostatus and self
  Participants expressed profound HIV-related internal stigma, which led to self-isolation and compromised engagement in HIV-related care during pregnancy. As this participant vividly describes:
*In my life, I was feeling I hated myself; I was suspicious that everybody knew my HIV status. I felt I was finished, I did not want to see people, and I spent much of the time in the house. Whenever I would see someone, I would think that this one knows things about me, I was always quiet and alone. I would not be outside, unless when I was going to hospital. -* (Participant #8, 5 months postpartum).
  However, the process of viewing HIV as an infection that affects many people and accepting her own HIV status was a powerful step towards self-acceptance. She continues:
*I denied myself, but later I consoled myself and I felt I would go through this. I consoled myself as a person and said that let me not become a laughing stock, good enough am not the first one and am not the last one [to have HIV] . It is a usual thing; let me accept*. - (Participant #8*)*.
  Another participant explained that starting ART initiated her process of accepting her HIV status and herself, underscoring the importance of self-acceptance as a fundamental step towards coping with the challenges of being a pregnant WLWH.
*Before I started ARVs, I was not well, physically, and my CD4s were low. I would feel rejected among my friends. However when I started ARVs I felt strong, and I said ‘why don’t I have a baby?’ I had good health, I came to like myself, I cooked food, I ate it, and I was happy. I was not thinking that HIV is even there. I accepted myself. I felt like HIV was like any other illness like malaria or just a minor accident you can get while taking a walk. I felt that strong.* - (participant #6 8 months postpartum).
2. Individual level: Self-reliance
  Several participants described experiences of partners denying responsibility for the pregnancy and abandoning the relationship. Without financial support from partners, participants explained how they relied on their own skills and assets to provide for themselves and their babies. The following participant explains how difficult her relationship with her partner was and what she went through to provide for herself and the baby.
*There is no good relationship with my partner because he has a second wife. When he saw that the children were dying, he said …let me bring another wife, the children you will have could also die. Therefore, he brought another wife and she has two children… This time when I told him that I was pregnant, he did not mind at first but when I was 8 months, he said, it is not mine; you know where you will put it [the baby]. I said, ok no problem; I will cater for myself….I felt my heart telling me, after all, I know how to work for myself, I must look for how to survive, look for money to make sure that after delivery I get food to eat. I ran up and down, did day labor , got money, kept it, bought my things for carrying the baby, I prepared myself and packed until I got into labor and came to hospital*.- *(*Participant #11, 12 months postpartum)
  Another participant whose partner did not want her to have more children because of her HIV status recounted the experience of her partner denying the pregnancy and his responsibility to care for her and their child. She then describes how she coped through self-reliance and a confidence in her own abilities.
*When I told him [about the pregnancy], he [partner] quarrelled, and he kept quarrelling, but later … he was like of course you know how you are [HIV+]; you would stop there [having children]. You do it again [get pregnant] you will be damaging your life. Don’t think that [I] am stopping you from having children but you should know what you are. Now when I saw that he was going to stress me (… they keep, teaching us here [clinic] that when you stress yourself you lose CD4 and you will be reducing on your life), I gave up on him as a person. I knew I would not be stranded like failing to get what to put on, what to eat or what to clothe the baby; I was not scared about those because I knew I would find them. Whether the job is there or not, can a person insist and remain on one thing forever? If I lost the job, I would look for something else to do. I have my two hands.-(*Participant #5, 5 months postpartum)
3. Interpersonal level: Support from partners, families, and friends
  Some participants described that receiving support from their partners and other family members positively impacted their pregnancy experience and postpartum outcomes. This participant described how her partner committed to helping her adhere to ART and PMTCT protocols so they could have an HIV-uninfected baby.
*He (partner) was not OK [with the pregnancy] initially, though he wanted to have a child, he was also scared like me, and he was even scared more. … in the end he decided that we should not abort the baby… He promised that he would be helping me, reminding me, go to the clinic and take my medications, to make sure that I follow all these steps and see what will come out. He did more than me (laughs) because even bringing the child to the clinic, he is the one who decided and kept on monitoring, is he taking medicine, are you taking him to the clinic, can I have a look at the papers, he was more serious than I was. - (*Participant #4 woman, *6 months postpartum).*
  Some of the participants relied on family members and friends for financial support to take care of themselves and their children. This woman discusses how friends and family members provided financial and other forms of tangible support.
*When I gave birth, they [community members] helped me a lot, people gave me food, and I did not suffer at all. Even the 20,000 [Ugandan shillings] I had kept, I bought clothes for the baby. Now am fine, I have no problem. I get support from people, some give me clothes, such things, when am stuck with no school fees and transport to hospital, they help me with the money and I pay them later …. I do not get stressed; I befriended those who love me. Those who help me are also sick [HIV+], so we know each other in such problems. In addition, my sister and my brother also help me. (*Participant #12 woman, 9 months postpartum*)*
  Support from friends, especially those also living with HIV, helped women identify hope within their pregnancy experience.
*I first felt bad and asked why I was going to give birth to those to leave behind [after death]. However, I have a friend I talked to; she has been on medicine [ARVs] for long. She told me, “You want to tell me that all these people you see…You can even produce 8 children, when you are still alive, the children will grow and get married when you are there [alive]. Don’t lose hope” that is how I gave up on thoughts and worries. - (*Participant #15, 20 months postpartum).
4. Organizational level: HIV-related healthcare delivery and system supports
  For some women, the support of trusted healthcare providers regarding PMTCT practice helped women overcome fears of perinatal transmission, engage in HIV care, and have an HIV-uninfected child.
*After realizing that my second child was the way I am [HIV-positive], I felt that I should try to have another child. … I was scared but at the same time, I wanted to have one [HIV-negative child]. … I wanted a child, so I decided to accept and follow whatever the health care providers told me and make sure I get a child who is free [HIV negative]. Therefore, I decided not to stress myself … You know after accepting, there was nothing much, because I made sure I took my medicine, came for antenatal care, and things were normal*. - *(*Participant #4 woman, 6 months postpartum)*.*
  Observing other women at the HIV treatment clinic who had successful pregnancies helped women to find strength to cope with the fears and challenges of their own pregnancy.
*I got so many problems… Because they tested me and found that I was HIV positive, I asked myself, ‘will I deliver the baby and it survives or am I going to suffer with this pregnancy or die?’ However, when I kept getting UARTO staff [HIV clinic] comforting me and telling me to take my medicine properly I got hope. Whenever I would go to hospital and see other women with their children who are healthy [HIV negative] yet they are on medication, I became strong. I stopped thinking and worrying, I was as if I had no virus, I ate and drank and I became strong. - (*Participant #20, 18 months postpartum*.*
5. Community level: Support from church community and spirituality
  Support from the church community and personal religious beliefs and spirituality helped some women manage challenges. This participant shared her experience of how she had considered suicide on several occasions but started a process of recovery with support from prayer and her church community.
*I was thinking a lot, I would think about this pregnancy, “Oh my God why did I do it, [get pregnant]” and then I would curse the man. I woke up one morning I went to throw myself in the river, [but] when I saw how the water was moving, I walked back home. I was thinking, the man had left me, I did not have money, I am HIV positive and I did not have anyone to turn to. Later I said, “Let me be patient, God will take care of me”. So whenever I would feel bothered, I would pray and I said let me hand myself to God. It helped me, because if I had not become born again [religion], I would have killed the baby and myself. However, whenever I would go to church, and I see how they pray and preach I would feel touched. I saw myself getting better and I saw that God could work. -* (Participant #8, 5 months postpartum)
  Another participant had misunderstandings with the partner who wanted to take the child away from her following their separation but she noted that she coped through prayer and the support of her church community and in the end, managed to stay with her child.
*Before I got pregnant, we had no problem at all. However, after giving birth, he behaved extremely badly towards me. …He disturbed me a lot to the point that he no longer wanted me to work. He refused to provide support for the child and wanted to take her [child] to the other wife. He [partner] gave me many troubles. I would say that this man has killed me [infected her with HIV] and now he is making me suffer. I prayed the rosary and dedicated myself to the Virgin Mary to help me through these troubles and God helped me through. I would ask people in the village to pray for me so that my child is not taken away from me. I offered all that to God, he helped me through, in the end I was allowed to stay with my child.* - *(*Participant #19, 21 months postpartum*)*.

## Discussion

Our study highlights positive coping strategies WLWH employed to manage challenges encountered during pregnancy and postpartum period in a rural African setting. The findings suggest that with positive coping strategies women WLWH manage extreme social and psychological challenges and have positive pregnancy outcomes.

Consistent with previous studies, we found that acceptance of HIV status and acceptance of self was a primary coping strategy used by women. Acceptance of HIV status and acceptance of self helps individuals to reframe their situation [51, 52] and is also a sign of motivation to deal with difficult situations, overcome denial and face reality [53].

Our study findings showed that some participants who had limited support from their partners became self-reliant to provide for themselves and their child. This finding is consistent with previous work showing that economic stability helps women with HIV to cope positively and overcome HIV-related stressors [54, 55]. Although women are able to cope and are important actors, they should not be saddled with all the responsibility. Partners, community members, family members and health care providers, and a larger structural environment should also play a role in alleviation of the challenges. After all, the role of partner and family and community support has been documented to reduce the risk of psychological distress among WLWH [9–11, 56]. In addition to helping WLWH cope , social support enables women to gain useful information about their health and availability of health care services [57–60]. Importantly, social support has been shown to protect against HIV disease progression [10, 11, 61] leading to longer survival among persons with HIV [62].

Some of our participants reported that support from partners and other family members helped them navigate pregnancy-related aspects of HIV including ART adherence. However as documented by Udobong and colleagues [63] partners are not always supportive and many tend to blame the women for having brought HIV to the family and abandon them.

Some women relied on family members for tangible and intangible support to cope with the stressors of being pregnant and living with HIV. These findings are in line with previous research highlighting that family support is associated with reduced stress, improved medication adherence, and reduced risks of psychological distress leading to improved quality of life among HIV positive women [52, 63–66]. Moreover, studies have shown that family support improves physical outcomes and protects against negative social consequences of living with HIV [9, 10]. This finding differs from what was found in Nigeria where WLWH reported that they were often abandoned by their families after disclosing their HIV status and this limited their access to medical care and led to disease progression [63].

Our findings also indicated that support from healthcare providers regarding PMTCT practice helped women overcome fears of perinatal transmission, engage in HIV care, and thus have HIV-uninfected children. This is in agreement with a previous study in Uganda where women attending PMTCT clinics described health workers as being caring and supportive, which helped women to deal with challenges and fears related to an HIV positive diagnosis [67].

Participants were able to cope and gained strength by observing other women who had gone through similar experiences. This is related to what has been documented in a previous study that knowing someone who is living with HIV enables those infected to accept their HIV status because of the inner comfort they feel knowing that someone shares their experience [25, 68]. This finding echoes earlier work showing that peer support groups play an important role in helping women living with HIV to understand HIV and PMTCT programs leading to retention in care and adherence to ART [69–72]. On the other hand participants in our study did not feel comfortable disclosing their status to community members for fear of stigma and ridicule contrary to what has been reported in a previous study that community members play a role in helping WLWH adhere to their medication and cope with the illness [70].

Some of our participants leaned on involvement with the church, spirituality, and prayer to cope with the stressors of pregnancy and the postpartum. This finding is consistent with previous research that religious involvement is common among HIV positive pregnant women and has been noted to be an important factor for adherence to medication and engagement in PMTCT care [52, 54, 73, 74]. In a South African study among people recently diagnosed with HIV, Myint [51] showed that religious involvement helped individuals find new meaning in life, obtain spiritual support, and cope with the psychosocial effects of an HIV diagnosis. Use of religion as a coping strategy has also been reported in previous studies to be an important source of strength and meaning to life for women living with HIV [34, 51, 52].

A previous study in Uganda revealed spirituality to be an important strategy for WLWH to cope with HIV-related stigma, with women expressing that while they were often stigmatized in the general community, a belief in “God’s acceptance” empowered them to persevere and find hope for the future [75]. Consistent with previous studies, we similarly found that religious involvement and prayer played an important role in helping WLWH cope with the challenges of pregnancy and the postpartum period [76–79]. Our findings differ from findings by Trevino and colleagues [34] who noted that spiritual struggle affects the ability of HIV positive individuals to cope with their illness.

The study had limitations that included a small study sample that may limit generalizability of the study findings to all HIV positive women during pregnancy and the postpartum period. Secondly, we interviewed women with pregnancy in the previous 2 years, which meant most of the women had navigated the pregnancy and the postpartum period and had adapted ways to cope with the challenges associated with pregnancy. The findings may also be limited by recall bias since we interviewed women about their pregnancy and postpartum experiences several months after childbirth a fact that could have had an effect on the recollection of their experiences of pregnancy and childbirth. We also did not do our analysis by depression scores given the small sample size and the fact that coping strategies were not something we explored in our interview. Finally, participants that were enrolled in UARTO received quarterly follow-up care, with reimbursement for transportation and as such, they may not be a typical representation of women living with HIV and engaged in HIV care.

## Conclusions

Despite availability of ART and PMTCT care, HIV positive pregnant and postpartum women face numerous psychosocial challenges that are not addressed in HIV or antenatal clinics. Community- and clinic-based programs for WLWH must acknowledge the myriad psychosocial stressors acting on the health and well-being of pregnant and post-partum women and incorporate psychosocial care within their care programming. Although WLWH are able to cope with challenges there is a need to encourage WLWH to form support groups through which they can encourage each other as they navigate HIV care. Moreover, previous research has documented that WLWH who are in peer support groups adhere to the HIV treatment cascade which improves physical and emotional outcomes [68–70]. Health care providers serving pregnant WLWH may help minimize psychosocial challenges and psychological distress by working to support, promote, and facilitate positive strategies for coping that women are already using. These efforts include regular screening of patients for psychological distress in the HIV clinics and refer for mental health assessment and treatment.

Community education and sharing information on how different WLWH cope with challenges may be beneficial to other women who are grappling with similar challenges.

Additionally policies that support networks of WLWH should be put in place and funding support should be provided through existing funding mechanisms in order to respond to the needs and challenges of WLWH. Local support networks in Mbarara like The AIDS Support Organisation (TASO) and other programmes that support WLWH for economic empowerment and improved livelihoods should be strengthened. Finally networks that aim to support WLWH including The AIDS Support organisation (TASO), International community of WLWH and AIDS in Eastern Africa (ICWEA), MAMA’s CLUB which is a psychosocial support group for HIV positive mothers, the National coalition of women with AIDS in Uganda and Global Coalition of Women against AIDS in Uganda should be strengthened and supported to expand across all HIV clinics to benefit all WLWH.

## Additional files

Additional file 1: Interview guide: Postpartum and antenatal depression among women enrolled in Uganda AIDS Rural Treatment Outcomes Study (UARTO): Patient Participant Interview Guide. (DOCX 26 kb)

Additional file 2: Codebook: Postpartum Depression (PPD) Participant Interviews Codebook. (DOCX 30 kb)

## Abbreviations

ART: Antiretroviral therapy
HIV: Human immunodeficiency virus
HSCL: Hopkins Symptom Checklist
PMTCT: Prevention of mother-to-child transmission of HIV
UARTO: Uganda AIDS Rural Treatment Outcomes
WLWH: Women living with HIV
