# Supplementary material for: Understanding coping strategies during pregnancy and the postpartum period: a qualitative study of women living with HIV in rural Uganda
Source: BMC Pregnancy Childbirth. 2017 May 8;17:138. doi: 10.1186/s12884-017-1321-9 (PMC5423027; doi:10.1186/s12884-017-1321-9)
Supplement: Supplementary file 2 — Codebook: Postpartum Depression (PPD) Participant Interviews Codebook. (DOCX 30 kb) [file 12884_2017_1321_MOESM2_ESM.docx]

**Postpartum depression Participant Interviews Codebook**

**Challenging aspects of pregnancy/childbirth/postpartum for HIV infected women** *[Code text in which women talk about the challenges – explicitly or implicitly – of their pregnancy experience]*

- Child loss / sickness/ child HIV status *[Text in which women talk about child loss, child sickness in the context of her pregnancy experiences since HIV+]*
  - - - *Concerns*
      - *Actual experiences*
- Child neglect (physical abuse) *[instances of hitting child, neglecting basic needs of the child]*
- Health care for children *[challenges related to accessing clinics, challenges related to child care, difficulties in general HIV treatment and in maternal health care]*
- Gender of child *[disappointment/frustration about the gender of baby]*
- Maternal health / PMTCT
  - - - Physical (*opportunistic infections, weight loss, CD4 count*)
      - Symptoms of depression
        - Crying
        - Hopelessness
        - Suicidal ideation
        - Difficulty sleeping
        - Guilt
        - Psychosomatic symptoms (back pain, headache)
        - Self-loathing
      - Prioritizing child health over maternal health
      - Challenges accessing / following up with healthcare ) *[Challenges of following up with ANC visits, postpartum visits, breastfeeding recommendations, testing, negative reactions from HCW re pregnant and HIV+ (e.g. 004)]*
      - Healthcare costs associated with being infected HIV+ *[transportation to the clinic and hospitals, regular visits with HCW, additional food to take medication]*
      - ARV adherence *[Challenges of adhering to ART in context of clinical sxs of preg (e.g. nausea) or postpartum (tired, feeding new baby, hard to organize life]*
      - Breastfeeding practices *[Challenges of adhering to breastfeeding practices/stigma of not breastfeeding/costs of milk, stress of breastfeeding knowing that there is risk of transmission (anxiety relief after breastfeeding is finished)]*
      - Challenges related to evolution over time of PMTCT recommendations/practices
      - Physical/Psychological Safety *[experiences related to unsafe housing or community, stigma around HIV, poverty etc. that contributes to psychological distress]*
      - HIV testing *(text about timing of HIV testing relative to referent preg – e.g. new diagnosis)*
- Financial *[Financial responsibilities that come with pregnancy (e.g. increased nutritional needs, harder to work) and having children – feeding, clothing, school, increased transport costs for more healthcare visits, How to find a job, child care while working, food and housing insecurity, custody disputes, relying on partner economic support* “I told my mother let me go back because if I stay here when am sick, how will that help me, am I really going to leave this world and leave nothing in it. She said “it’s up to you but me I had not want you to go back to the man because he does not care for you and your brothers had decided to take care of you and give you everything you want” (11*]*
- Partners *[Text in which women talk about partner challenges related to pregnancy – e.g. Refuse to provide transport to clinic, IPV, accuse her of having someone else’s child, etc.]*
  - - - Intimate partner violence
      - Experiences with partner’s other partners (*co-wives, other sexual partners*)
      - Uninfected partner/Partner Status (*challenges related to being in a serodiscordant relationship, in reference to this pregnancy only*)
- Positive
- Negative
- Don’t know
- Reproductive autonomy *[experiences related to family planning, negotiations around sex and conception, use of contraception]*
- Abortion *[experiences of abortion in clinic, attempts to terminate pregnancy outside of medical environment, decisions related to desires to abort baby, discussion with partner about abortion as an option]*
- Structural barriers to women as mothers *[Custody fights with partner if couple is no longer together, limited access to material resources]*
- Fertility Concerns *[text where participants describe issues related to getting pregnant, miscarriages, and other challenges related to fertility]*
- Stigma of HIV+ and pregnancy/childbearing *[discouraged to reproduce because of status – if this is in the clinic, put under healthcare node, job insecurity related to HIV stigma, concerns about public disclosure, confusion about infection]*
- Disclosure as negative experience *[to community, friends, social network e.g. 004 does not want to disclose]*
- Family disclosure as it affects the referent pregnancy *[Distancing the participant from family due to HIV stigma, hiding status from family for fear of social exclusion]*

**Positive aspects of pregnancy/childbirth/postpartum for HIV infected women** *[benefits of reproduction, experiences of fulfillment of gender role through motherhood, positive feelings about parenting]*

- Healthy baby *[baby who is HIV- brings relief about HIV anxiety to mother, strong/healthy children represent good parenting and ability to provide for the baby “* Six weeks, so like one month and two weeks. Whenever I would go to pray I would say, “oh God, can’t my child survive this, if am sick, can’t they test my child and they find that he is fine.” Whenever I would pray, I would pray for that thing. So when the time arrived and I came, they drew blood from him and I went, and after I don’t know what period, when I brought him back at the clinic, when the nurse told me that, ‘the first blood of your child is ok,’ I felt so grateful, I became happy”(8)*]*
- Developing a relationship with child *[establishing a mother-child relationship that is fulfilling to participant, child brings great pleasure to mother]*
- Health care for children *[Ease and satisfaction in accessing clinics, help with child care, good advice related to parenting/resources, health care providers who are able to communicate knowledge and act as references for mothers]*
- Gender of child *(e.g 004 is happy to be having a boy as part of her experience)*
- Maternal Health *[access to services (HIV or other) through antenatal care, benefits to health through pregnancy]*
- Health care system *[supportive clinicians and health care workers through pregnancy and child birth, financial/material help with child associated costs through health care system; pmtct empowers women to protect her baby]*
- UARTO RA’s *[* *relationships developed with UARTO RA’s that provide material, educational, psychological or social support, access to care or any other form of support that exists though the UARTO structure from study staff* *“*But when I found this (name of RA), when I related to her, all the things, all the things she is the one that gave them to me, the bed sheets, the cloths, I really thanked God, I don’t know, I kept saying that what will I do? But she gave me bed sheets, and she told that other one who is pregnant (another RA that was in the UARTO building at the time that I conducted the interview), she also gave me cloths*”]*
- Fulfilling reproductive roles*[satisfying expectations that women should reproduce]*
- Reproductive autonomy *[experiences related to family planning, negotiations around sex and conception, use of contraception]*
- Relationship with partner *[conceiving/delivering a child brings the couple together, strengthens relationship through fulfillment of reproductive roles, anchors partners to one another in caring for the child]*
- Community support *[material/financial support from people in the community to raise the child, help with child care, medical access through community members* (8)*]*
- Disclosure as positive experience *[acceptance from family/friends/community of HIV + status, access to necessary services and support]*
- Family disclosure as it affects the referent pregnancy *[Disclosure as a way of gaining social support]*

**Relationship with Partner** *[participant experiences and feelings towards partner, examples of partnership dynamic and themes of support or lack of support]*

- Relationship Characteristics *[Sociodemographics of the relationship -- how long together, marital status, living status, aspects of relationship that explain partner expectations of one another]*
- Cohabitation
  - Always/mostly
  - Sometimes
  - Rarely
  - No
- Marital Status
- Length of Partnership *[how long the couple has been together]*
- Relationship Roles *[Male productive role + female reproductive role, examples of partner taking responsibility to support family economically, especially during pregnancy, participant perception of advantages/disadvantages of a partner with or without]*
- Other Partners *[participant or partner]*
- Disclosure *[participant has disclosed HIV status to partner]*
- How partner serostatus affects the relationship *[Knowledge/education of HIV from HIV negative partner is a reflection of the investment in relationship and care for the participant, precautions taken to limit risk of transmission to partner, code using this node outside of referent pregnancy]*

**Attitudes towards the Referent Pregnancy** *[did the participant want to get pregnant? Conception strategies, examples of family planning, desires (or lack of desire) to have baby]*

Participant

- Wanted *[participant wanted to conceive and have a baby]*
- Not-wanted *[participant did not want to conceive and have a baby]*
- Planned *[planning strategy used to conceive, mother eager for pregnancy]*
- Not-planned *[no family planning strategy used, pregnancy unexpected]*
- Ambivalent/unclear *[e.g. once pregnancy happened, child accepted but not really clear that child was wanted (or not wanted), planned (or not planned)]*

Partner of referent pregnancy

- Wanted *[partner wanted to conceive and have a baby]*
- Not-wanted *[partner did not want to conceive and have a baby]*
- Planned *[planning strategy used to conceive, partner eager for pregnancy]*
- Not-planned *[no family planning strategy used, pregnancy unexpected]*
- Ambivalent/unclear *[e.g. once pregnancy happened, child accepted but not really clear that child was wanted (or not wanted), planned (or not planned). E.g. 004 says of partner “he wanted a child of course but he also felt ‘uh, we are going to have another sick child, what is going to happen?”]*

**Coping Strategies** *[Strategies that women use to cope with stressors of pregnancy/postpartum/childbirth]*

- Denial *[Denial of HIV+ status, refusing treatment, repeated HIV tests, avoiding clinic/pharmacy]*
- Acceptance [e.g. 004 accepts hiv status, engages in care, seems to be empowered by adhering to treatment strategies and by managing infection autonomously, mental health benefits of active acceptance of self ]
- Religion *[Experiences of prayer, divine intervention and broader teleological perspective. Church as social support?* “I would pray, if I would go to the church and I spend the day in King of kings on Mondays, we usually spent the day there and came back at 6 Pm. When I would go and pray and I listen to how they preach, sometimes if I would see people giving testimonies, I would feel that it is not mine alone” (8)*]*
- Family *[Family pressure to reproduce, family pressure not to reproduce, family support w/ HIV status and with child rearing/ through pregnancy, choices made between family and children]*
- Becoming more independent/self reliant *[Examples of economic independence out of necessity to provide for the child, experiences of raising the child without partner, developing strategies to create steady flow of income* “In the plantation there has really been a change. I work before it would be bushy, but I work, I don’t touch their bananas. There is there brother who harvests them, when they steal them, I give them the report, in fact recently they have arrested them, and they brought a spy who arrested the ones that were cutting them. In fact recently they were here. They like me so much and even the villagers tell him that since you have been bringing workers, now you have really brought. There is nowhere that I go, I don’t roam about, if am not home I have gone to the church or like I have come here. I don’t go to the trading center to roam about, so that is why they liked me” (*8)]*
- Normalization of HIV infected women having children *[developing awareness of how common pregnancy is for positive women, association with women in the community who have similar experiences, examples of successful PMTCT]*
